# PrEPped for COVID? The association between HIV-PrEP use and COVID-19 among men and gender diverse people who have sex with men: findings from three large online surveys in the United Kingdom

**DOI:** 10.1186/s12889-025-26127-0

**Published:** 2026-01-22

**Authors:** Caisey V. Pulford, Holly Mitchell, Jack RG. Brown, Alison R. Howarth, David Reid, Hamish Mohammed, Gwenda Hughes, Catherine H. Mercer, John Saunders

**Affiliations:** 1 Blood Safety, Hepatitis, STIs and HIV Division, United Kingdom Health Security Agency (UKHSA), 61 Colindale Avenue, London, NW9 5EQ UK; 2 The National Institute for Health & Care Research Health Protection Research Unit in Blood Borne & Sexually Transmitted Infections at University College London in partnership with United Kingdom Health Security Agency, London, UK; 3 Institute for Global Health, University College London, London, UK; 4 Department of Infectious Disease Epidemiology, London School of Hygiene & Tropical Medicine, London, UK

**Keywords:** HIV, PrEP, COVID-19, GBMSM, Survey

## Abstract

**Background:**

Oral tenofovir and emtricitabine taken as pre-exposure prophylaxis (HIV-PrEP) is highly effective at reducing Human Immunodeficiency Virus (HIV) acquisition. Nucleotide analogues have also been shown to inhibit Severe Acute Respiratory Syndrome Coronavirus 2 (SARS-CoV-2) polymerase activity in vitro, raising the question as to whether tenofovir-based HIV-PrEP could be useful for the prevention and treatment of COVID-19. We sought to examine the association between HIV-PrEP use and self-reported COVID-19 testing outcomes and respiratory symptoms among men and gender diverse people who have sex with men in the UK.

**Methods:**

Participants completed online surveys deployed at three time points during the COVID-19 pandemic (Survey 1 (S1) 23/06/20 − 14/07/20; S2 23/11/20 − 12/12/20; S3 23/03/21 − 14/04/21), including men (cis/transgender), transwomen or gender-diverse people reporting sex with another man (cis/transgender) or non-binary person assigned male at birth. The outcome was self-reported COVID-19 testing and COVID-19 related respiratory symptoms including a new continuous cough, high temperature or loss of smell and/or taste during the most recent lookback period. All participants reporting taking HIV-PrEP in the past year were compared with those who did not. Analysis was performed using logistic regression, adjusting for sociodemographic and behavioural factors.

**Results:**

Among 4,544 participants living without HIV across three surveys (89.7% of all survey respondents), 21.4% (*n* = 970) reported recently using HIV-PrEP, 564 (12.4%) participants reported respiratory symptoms and 160 (3.5%) reported a positive COVID-19 test. There was a slight, non-statistically significant positive association between HIV-PrEP use and a COVID-19 positive test in the first survey-period (P1) (adjusted Odds Ratio (aOR) = 1.11, CI = 0.51–3.98). This positive association significantly increased across survey periods (P2: aOR = 4.26, CI = 1.21–14.97, P3: aOR = 9.02, CI = 2.69–30.31). There was a positive association between HIV-PrEP use and respiratory symptoms in the first survey-period (aOR = 1.45, CI = 1.03–2.03). However, this positive association reversed in survey-period 2 and 3 (P2: aOR = 0.62, CI = 0.40–0.95, P3: aOR = 0.45, CI = 0.28–0.72).

**Conclusions:**

We found no evidence that HIV-PrEP use protected against COVID-19 among participants. Conversely, we identified a positive association between HIV-PrEP use and COVID-19 which may reflect uncaptured behaviours that increase COVID-19 exposure amongst those using HIV-PrEP. The decline in respiratory symptoms in HIV-PrEP users over time might reflect some immune protection following exposures to respiratory viruses through more social mixing or a direct impact of HIV-PrEP on symptom severity.

**Supplementary Information:**

The online version contains supplementary material available at 10.1186/s12889-025-26127-0.

## Background

In March 2020, shortly after the World Health Organisation (WHO) declared COVID-19 a pandemic, a national lockdown was implemented in the United Kingdom (UK) [1, 2]. This mandated that people ‘stay at home’, meeting people from outside their household or ‘social bubble’ was not allowed, and certain businesses were closed in an attempt to slow down the transmission of Severe Acute Respiratory Syndrome Coronavirus 2 (SARS-CoV-2). While the acute phase of the COVID-19 pandemic has now ended, it prompted important questions about how existing public health interventions might influence susceptibility to emerging infections.

Internationally, clinical trials were started to identify effective therapies for the prevention and treatment of COVID-19. Potential antiviral drug therapies included the antiretrovirals (ARV) lopinavir and ritonavir because of in vitro activity against the closely related coronaviruses - severe acute respiratory syndrome (SARS) and Middle East Respiratory Syndrome (MERS). However, there was limited evidence for clinical benefit [3–5]. Nucleotide analogues were also of interest as they showed inhibitory activity against SARS-CoV-2 polymerase in vitro [6]. Observational data among people living with HIV suggested that two other ARVs (tenofovir (TDF) and emtricitabine (FTC)) may have had a beneficial impact on infection rates and risk of hospitalisation following COVID-19 infection [7, 8]. For example, a study looking at 77,950 people living with HIV and receiving antiretroviral in Spain identified that patients specifically receiving TDF/FCT have a lower risk of COVID-19 and related hospitalisation than those receiving other therapies [7]. However, another study did not demonstrate an impact on COVID-19 seroprevalence or clinical manifestations among users of TDF/FTC as HIV pre-exposure prophylaxis (HIV-PrEP) [9].

In the UK, HIV-PrEP use among men and gender diverse people who have sex with men has increased significantly. In Scotland and Wales, national HIV-PrEP programmes started in 2017 and Northern Ireland started a pilot in 2018 [10]. In England, more than 24,000 people were provided HIV-PrEP through the Impact trial between October 2017 and October 2020 with routine commissioning thereafter [11].

HIV-PrEP has traditionally been studied in the context of HIV-prevention. However, the COVID-19 pandemic prompted new questions about whether HIV-PrEP use may have broader implications for other infectious diseases such as respiratory illnesses (such as COVID-19). Such studies have been conducted internationally, each focusing on their respective international contexts during the COVID-19 pandemic [9, 12–16]. Using data from the Reducing inequalities in Sexual Health’ (RiiSH)-COVID surveys we have also been able to explore this question in the UK. Specifically, we sought to examine the association between HIV-PrEP use and COVID-19 among men and gender diverse people who have sex with men during the first year of the pandemic in the UK, taking account of a range of sociodemographic and behavioural factors. This included (1) examining the association between HIV-PrEP use and self-reported COVID-19 testing outcomes and (2) examining the association between HIV-PrEP use and self-reported COVID-19 related respiratory symptoms.

This study has been important in considering previously unexamined potential of HIV-PrEP among men and gender diverse people who have sex with men (who make up the vast majority of PrEP users in the UK), and it remains relevant for improving our understanding of the wider health impacts of HIV-PrEP as a biomedical prevention strategy. These insights can inform more integrated public health approaches in the future.

## Methods

We conducted an observational study using data collected at three timepoints during the first year of the COVID-19 pandemic through the ‘Reducing inequalities in Sexual Health’ (RiiSH)-COVID study. RiiSH-COVID used repeat, cross-sectional, online community surveys that were self-completed by participants. The surveys were adapted from a survey conducted in 2017 [17, 18] and focused on understanding the impact of the COVID-19 pandemic on sexual behaviours and health service use among men and gender diverse people who have sex with men in the UK. In total, four RiiSH-COVID surveys were conducted, each of which was fielded for 2–3 weeks at key points during the pandemic. This study includes data from those fielded in the first year of the pandemic (RiiSH 1,2 and 3). The questions from each were largely comparable, with some questions being amended, added, or removed to reflect the changing landscape of the COVID-19 pandemic (for example questions relating to vaccination were only added in RiiSH-COVID 3).

### Setting and participants

Participants were recruited using advertisements placed on social networking sites (Facebook, Twitter, Instagram). In RiiSH 1, geospatial dating applications (Grindr, Hornet) were also used for recruitment. Eligible participants were UK-residents; aged 16 years or older; men and other people assigned male at birth regardless of gender identity; who reported sex in the last year with a cis or transgender man or other people assigned male at birth. Online consent was obtained from all participants and no financial incentives were offered. The denominator for these analyses was limited to those who reported living without HIV.

### Data collection

Survey questions referred to lookback periods which approximately corresponded to the three months immediately prior to the survey and asked about sexual relationships and behaviour, drug use, HIV-PrEP use, Sexually Transmitted Infections (STI)/HIV symptoms and testing, use of sexual health services. Additional questions related to COVID-19 including symptoms, testing, diagnosis and vaccination status.

The dates in which each survey was in field are as follows (note that dates of national restrictions/relaxations varied by a few weeks across UK countries). Survey 1 (S1) was in field between 23rd June 2020 and 14th July 2020, and asked questions focused on the period between the start of the first national lockdown (23rd March 2020) and the time at which participants took S1. An example of phrasing in the questionnaire is: ‘Thinking about the time since lockdown (23 March 2020), how often have you taken PrEP?’. This timeframe will henceforth be referred to as Lookback Period 1 (P1). Testing during P1 was limited to those conducted at test-sites for certain population groups.

Survey 2 (S2) was in field between 23rd November 2020 and 12th December 2020, and asked questions focused on the period between when the first lockdown was eased to minimal restrictions (July 2020) and the time at which participants took S2. An example of phrasing in the questionnaire is: ‘Thinking about the time since July 2020, how often have you taken PrEP?’. This timeframe will henceforth be referred to as Lookback Period 2 (P2). P2 coincides with the time in which lateral flow diagnostic tests were used in targeted settings in those with COVID-19 symptoms

Survey 3 (S3) was in field between 23rd March 2021 and 14th April 2021, and asked questions focused on the period when the second national lockdown was in place (January 2021) to the time at which participants took S3. An example of phrasing in the questionnaire is: ‘Thinking about the time since December 2020, how often have you taken PrEP?’. This timeframe will henceforth be referred to as Lookback Period 3 (P3). P3 includes the timeframe of a community testing programme and the launch of the universal testing offer (in which people were asked to test twice weekly). Vaccination was available during this timeframe, however roll-out was not complete for all age-groups.

### Data analysis

Data analysis was performed using Stata 15 [19].

The key measures were created as per the following definitions: The outcome of interest was either self-reporting having a positive COVID-19 test or as reporting respiratory symptoms. The measure for a positive COVID-19 test included all those who self-reported having a positive COVID-19 test in the most recent lookback period (antigen/antibody). The measure for respiratory symptoms included all those who self-reported having a new continuous cough and/or a high temperature and/or loss of smell and/or loss of taste during the most recent lookback period. Respiratory symptoms, in line with those defined at the time of the study as being associated with COVID-19, were looked at because testing for COVID-19 was not widely available at the early stages of the pandemic. The measure for HIV-PrEP use during COVID-19 included all those who self-reported taking any HIV-PrEP in the most recent lookback period. For validation, we compared this result with the responses of the question asking participants to indicate when they last took HIV-PrEP. The measure for frequency of HIV-PrEP use during lookback period was defined as follows: ‘No HIV-PrEP’ includes participants who did not report taking HIV-PrEP during the lookback period; ‘Frequently used HIV-PrEP’ includes participants who reported taking HIV-PrEP daily or 4–6 times a week during the lookback period; ‘Infrequently used HIV-PrEP’ includes participants who reported taking HIV-PrEP intermittently, on and off, event-based or only once during the lookback period.

Pearson’s chi-squared tests [20] were used for comparisons of participant demographics, exposures of interest and COVID-19 positive test/respiratory symptoms between the three surveys. The associated *P-*values are presented, with *p* < 0.05 used as the threshold for statistical significance.

Binary logistic regression was used to examine associations between HIV-PrEP use and (i) respiratory symptoms or (ii) having a COVID-19 positive test across the first year of the pandemic adjusting for sociodemographic and behavioural factors reported by participants (age, ethnicity, education, employment status, country of residence, whether living with partner, chemsex and number of new partners). There were a number of additional behavioural variables which could also have been used to provide an indication of risk, however these correlate highly with each other and including all would have led to overadjustment of the model. Thus, a small number of key behavioural factors were included. Changing sexual behaviours in HIV-negative men who have sex with men during the COVID-19 pandemic was previously explored [21]. We used multivariable logistic regression to model the association between HIV-PrEP use (main effect) and respiratory symptoms/COVID-19 confirmed by test (outcome variable), including an interaction with survey wave (main effect) to assess effect modification. Stratum-specific estimates were derived using linear combinations of the relevant coefficients. Specifically, the effect of HIV-PrEP was calculated by summing the coefficient for the survey wave and its interaction with HIV-PrEP use. This approach allowed us to evaluate whether the effect of HIV-PrEP varied across lookback periods.

## Results

### Demographics of study participants by survey

In total 5,066 men and gender diverse people who have sex with men completed at least one of the surveys. There is a possibility that some participants took part in more than one survey. An attempt to pseudonymously link participants across surveys 1 and 2 was able to deterministically match only 1.4% of participants.

There were 4,544 participants who reported living without HIV and were included in this study. Specifically, 1,815 participants were included from S1, 1,361 participants from S2 and 1,368 participants from S3. The majority (96.9%) of participants were cisgender men, however a small number of transmen (n=45), transwomen (n=21) and gender diverse people assigned male at birth (n=77) were included. We identified no significant difference in gender distributions between each survey (p=0.442).

There were minimal differences in demographic characteristics of participants across surveys (Table 1). Overall, the median age of participants was 39 years [interquartile range (IQR) 16–80 years; range 16–81 years]. Most participants identified as white (88.3%), were resident in England (84.3%) and were born in the UK (77.9%). These demographics were similar across the three surveys. Just over half of all participants were educated to degree level or above (57.5%) and approximately three quarters were employed at the time of taking the survey (76.6%). There were no differences in the level of education (p=0.395) or employment status (p=0.139) of participants across the three surveys. Approximately one third of participants reported living alone (34.2%) and one third reported living with (a) partner(s) (30.4%), while the remainder reported living with friends (17.9%) or family members (17.5%).

**Table 1:** Demographics of participants who completed one of three online surveys of men and gender diverse people who have sex with men in the UK during the first year of the COVID-19 pandemic^1^

Demographics	Survey 1^2^		Survey 2^3^		Survey 3^4^		Total		*P* value^5^
	*n*	%	*n*	%	*n*	%	*n*	%	
Gender									
Cisgender male	1,759	96.9%	1,311	96.3%	1331	97.3%	4,401	96.9%	0.442
Trans male	18	1.0%	14	1.0%	13	1.0%	45	1.0%	
Trans female	9	0.5%	10	0.7%	2	0.1%	21	0.5%	
Other gender	29	1.6%	26	1.9%	22	1.6%	77	1.7%	
Age at time of taking survey									
16–29	495	27.5%	401	29.5%	357	26.1%	1,253	27.6%	0.028
30–44	636	35.0%	509	37.4%	481	35.2%	1,626	35.8%	
45–59	524	28.9%	342	25.1%	383	28.0%	1,249	27.5%	
60+	160	8.82%	109	8.0%	146	10.7%	415	9.1%	
Ethnicity									
White	1,601	88.2%	1,181	86.8%	1,232	90.1%	4,014	88.3%	0.001
Black	25	1.4%	43	3.2%	22	1.6%	90	2.0%	
Asian	106	5.8%	63	4.6%	53	3.8%	222	4.9%	
Mixed or Other	83	4.6%	74	5.4%	61	4.5%	218	4.8%	
Country of residence during lookback period									
England	1,558	85.8%	1,135	83.3%	1,136	83.0%	3,829	84.3%	0.054
Scotland	139	7.7%	145	10.7%	135	9.9%	419	9.2%	
Wales	79	4.4%	61	4.5%	70	5.1%	210	4.6%	
Northern Ireland	39	2.1%	20	1.5%	27	2.0%	86	1.9%	
Place of birth									
UK	1,422	78.3%	1,039	76.3%	1,080	79.0%	3,541	77.9%	0.223
Other	393	21.7%	322	23.7%	288	21.0%	1,003	22.1%	
Education level during lookback period									
Degree & above	1055	58.2%	792	58.2%	766	56.0%	2,613	57.5%	0.395
Below degree	759	41.8%	569	41.8%	602	44.0%	1,930	42.5%	
Employment status during lookback period									
Employed	1,394	77.3%	1,017	74.7%	1,062	77.6%	3,473	76.6%	0.139
Not employed	410	22.7%	344	25.3%	306	22.4%	1,060	23.4%	
Living situation during lookback period									
Alone	623	34.3%	465	34.2%	465	34.0%	1,553	34.2%	0.022
Family	333	18.4%	229	16.8%	233	17.0%	795	17.5%	
Friends	300	16.5%	284	20.9%	229	16.7%	813	17.9%	
Partner	559	30.8%	383	28.1%	441	32.2%	1,383	30.4%	

1. The values and %s shown for all categories may differ slightly to those presented by Brown et al. 2022 [22] due to differences in the denominator value used in each paper. For clarity the analysis in this paper includes all participants who reported living without HIV who are cisgender male, trans male, trans female or other gender. The analysis performed by Brown et al. is restricted to cis-gender males and did not exclude participants based on HIV status

2. Survey 1 (S1) was in field between 23rd June 2020 and 14th July 2020. Lookback period 1 (P1) focused on the time between the first national lockdown (23rd March 2020) and the time at which participants took S1

3. Survey 2 (S2) was in field between 23rd November 2020 and 12th December 2020. Lookback period 2 (P2) focused on the period between when the first lockdown was eased to minimal restrictions (July 2020) and the time at which participants took S2

4. Survey 3 (S3) was in field between 23rd March 2021 and 14th April 2021. Lookback period 3 (P3) focused on the period when the second national lockdown was in place (January 2021) to the time at which participants took S3

5. Results of χ 2 tests

### Changes in HIV-PrEP use over the first year of the pandemic

To examine the impact of the first year of the COVID-19 pandemic on HIV-PrEP use, we explored trends in the proportion of participants reporting taking HIV-PrEP, including frequency of use (Table 2). The proportion of participants reporting HIV-PrEP use varied across different periods of the pandemic (p<0.0001), increasing from 17.1% in P1 to 25.4% and 23.0% in P2 and P3, respectively. The increase in HIV-PrEP use between P1 and P2/3 coincided with changes in the frequency of HIV-PrEP use reported across the pandemic (p<0.0001). Specifically, there was an increase in the proportion of participants who reported frequent HIV-PrEP use (4 or more times per week) from 7.4% in P1 to 16.5% and 16.0% in P2 and P3, respectively. The proportion of participants reporting infrequent HIV-PrEP use (3 or less times per week, or event based) remained more constant between pandemic periods (P1:7.4%, P2:8.9%, P3:7.2%).

**Table 2:** HIV-PrEP use, number of new partners and chemsex use reported in lookback period of one of three online surveys of men and gender diverse people who have sex with men in the UK during the first year of the COVID-19 pandemic.^1^

Exposures of interest	Lookback period 1^2^		Lookback period 2^3^		Lookback period 3^4^		Total		*P* value^5^
	*n*	%	*n*	%	*n*	%	*n*	%	
HIV-PrEP use during lookback period									
No HIV-PrEP use	1,498	82.9%	1,014	74.6%	1,053	77.0%	3,565	78.6%	< 0.0001
Used HIV-PrEP	309	17.1%	346	25.4%	315	23.0%	970	21.4%	
Frequency of HIV-PrEP use during lookback period ^6^									
No HIV-PrEP or did not answer question	1546	85.2%	1015	74.6%	1053	77.0%	3164	79.5%	< 0.0001
Frequently used HIV-PrEP	135	7.4%	225	16.5%	216	15.8%%	576	12.7%	
Infrequently used HIV-PrEP	134	7.4%	121	8.9%	99	7.2%	354	7.8%	
Number of new partners during lookback period									
0	1,162	64.0%	514	37.8%	672	49.1%	2,348	51.7%	< 0.0001
1–2	381	21.0%	370	27.2%	342	25.0%	1,093	24.1%	
3+	271	14.9%	477	35.0%	354	25.9%	1,102	24.3%	
Chemsex during lookback period									
No chemsex	1,767	97.4%	1,299	95.4%	1,334	97.5%	4,400	96.8%	0.002
Chemsex	48	2.6%	62	4.6%	34	2.5%	144	3.2%	

1. The values and %s shown for all categories may differ slightly to those presented by Brown et al. 2022 [22] due to differences in the denominator value used in each paper. For clarity the analysis in this paper includes all participants who reported living without HIV who are cisgender male, trans male, trans female or other gender. The analysis performed by Brown et al. is restricted to cis-gender males and did not exclude participants based on HIV status

2. Survey 1 (S1) was in field between 23rd June 2020 and 14th July 2020. Lookback period 1 (P1) focused on the time between the first national lockdown (23rd March 2020) and the time at which participants took S1

3. Survey 2 (S2) was in field between 23rd November 2020 and 12th December 2020. Lookback period 2 (P2) focused on the period between when the first lockdown was eased to minimal restrictions (July 2020) and the time at which participants took S2

4. Survey 3 (S3) was in field between 23rd March 2021 and 14th April 2021. Lookback period 3 (P3) focused on the period when the second national lockdown was in place (January 2021) to the time at which participants took S3

5. Results of χ 2 tests

6. Frequency of HIV PrEP use during lookback period was defined as follows: ‘No HIV-PrEP’ includes participants who did not report taking HIV-PrEP during the lookback period or did not provide an answer to the question (for this reason, the value is sometimes higher than that given for no HIV-PrEP use reported above); ‘Frequently used HIV-PrEP’ includes participants who reported taking HIV-PrEP daily or 4–6 times a week during the lookback period; ‘Infrequently used HIV-PrEP’ includes participants who reported taking HIV-PrEP intermittently, on and off, event-based or only once during the lookback period

To understand whether there were any changes in key behavioural factors of participants across the first year of the pandemic, we assessed differences in chemsex and the number of new partners reported in each survey (Table 2). Participants who reported using HIV-PrEP reported having a greater number of partners than participants who did not report taking HIV-PrEP. However, there were differences in the number of new partners reported at different periods of the pandemic (p<0.0001), with the proportion of participants reporting no new partners halving between P1 (64.0%) and P2 (37.8%), before increasing in P3 (49.1%). Those reporting more than three new partners doubled between P1 (14.9%) and P2 (35.1%), before decreasing slightly in P3 (25.9%). A similar trend occurred in the proportion of participants who reported engaging in chemsex in each period, increasing between P1 (2.6%) and P2 (4.6%) before dropping in P3 (2.5%).

### COVID-19

We investigated trends in participants reporting experiencing symptoms of a respiratory infection or a positive COVID-19 test result in different periods of the pandemic (Table 3). The proportion of participants who reported experiencing respiratory symptoms decreased marginally across the pandemic from 13.6% in P1 to 11.8% and 11.4% in P2 and P3 respectively, although this change was not found to be statistically significant (p=0.109). In contrast, the proportion of participants reporting a positive COVID-19 test result increased significantly across surveys (p<0.0001). Specifically, 0.9% of participants reported a positive test result in P1, increasing to 3.7% and 6.9% of participants in P2 and P3, respectively.

**Table 3:** COVID-19 test/respiratory symptoms reported in each lookback period of one of three online surveys of men and gender diverse people who have sex with men in the UK during the first year of the COVID-19 pandemic

Outcome	Lookback period 1^1^		Lookback period 2^2^		Lookback period 3^3^		Total		*P* value^4^
	*n*	%	*n*	%	*n*	%	*n*	%	
Respiratory symptoms during lookback period									
Respiratory symptoms	248	13.6%	160	11.8%	156	11.4%	564	12.4%	0.109
No respiratory symptoms	1,567	86.3%	1,201	88.2%	1,212	88.6%	3,980	87.6%	
COVID-19 positive test during lookback period									
COVID-19 positive test	16	0.9%	50	3.7%	95	6.9%	160	3.5%	< 0.0001
No COVID-19 positive test	1,799	99.1%	1,311	96.3%	1,274	93.1%	4,384	96.5%	

1.Survey 1 (S1) was in field between 23rd June 2020 and 14th July 2020. Lookback period 1 (P1) focused on the time between the first national lockdown (23rd March 2020) and the time at which participants took S1

2. Survey 2 (S2) was in field between 23rd November 2020 and 12th December 2020. Lookback period 2 (P2) focused on the period between when the first lockdown was eased to minimal restrictions (July 2020) and the time at which participants took S2

3. Survey 3 (S3) was in field between 23rd March 2021 and 14th April 2021. Lookback period 3 (P3) focused on the period when the second national lockdown was in place (January 2021) to the time at which participants took S3

4. Results of χ 2 tests

### The association between recent HIV-PrEP use and respiratory symptoms

We investigated the association between HIV-PrEP use and respiratory symptoms at different periods of the pandemic, to account for differences in availability of COVID-19 testing throughout the pandemic. Of those 970 study participants in the three surveys reporting use of HIV-PrEP, 13.5% (n=131) reported respiratory symptoms and 86.5% (n=839) reported no respiratory symptoms when asked about the most recent lookback period to the survey they were completing. Of the remaining 3,565 study participants who reported no use of HIV-PrEP, 12.1% (n=431) reported respiratory symptoms and 87.9% (n=3,134) reported no respiratory symptoms when asked about the most recent lookback period to the survey they were completing.

After adjusting for sociodemographic and behavioural variables, we identified no significant association between HIV-PrEP use and respiratory symptoms (adjusted Odds Ratio (aOR=1.14, Confidence Interval (CI)=0.92–1.40.92.40, p=0.236) (S1 Table). However, subsequent analysis indicated evidence of an interaction, such that the magnitude of the effect of HIV-PrEP use on having respiratory symptoms changed significantly in each lookback period (interaction p=0.014). The results from the analysis including the interaction term are presented in Table 4. In P1 participants who had recently used HIV-PrEP were more likely to experience respiratory symptoms than those who had not used HIV-PrEP (aOR=1.45, CI=1.03–2.03.03.03, p=0.033). However, as the pandemic progressed this association reversed. In P2 and P3 participants who reported recent use of HIV-PrEP were less likely to experience respiratory symptoms than participants who had not used HIV-PrEP (P2: aOR=0.62, CI=0.40–0.95.40.95, p=0.029; P3: aOR=0.45, CI= 0.28–0.72.28.72, p=0.001).

**Table 4:** The association between respiratory symptoms (outcome) and HIV-PrEP use among men and gender diverse people who have sex with men during three study periods during the first year of the pandemic (main effects, including interaction term)

	No respiratory symptoms	Respiratory symptoms	Total	adjusted Odds Ratio^1^	95% Confidence Interval	*P* value
HIV-PrEP use in lookback period (interaction term included between these two main effects)						
No HIV-PrEP in lookback period 1	1,308 (87.3%)	190 (12.7)	1,498	1	-	-
Used HIV-PrEP in lookback period 1	253 (81.9%)	56 (18.1%)	309	1.45	1.03–2.03	0.033
No HIV-PrEP in lookback period 2	899 (88.7%)	115 (11.3%)	1,014	1	-	-
Used HIV-PrEP in lookback period 2	301 (87.0%)	45 (13.0%)	346	0.62	0.40–0.95	0.029
No HIV-PrEP in lookback period 3	927 (88.0%)	126 (12.0%)	1,053	1	-	-
Used HIV-PrEP in lookback period 3	285 (90.5%)	30 (9.5%)	315	0.45	0.28–0.72	0.001
Age at time of taking survey						
16–29	1,066 (85.1%)	187 (14.9%)	1,253	1	-	-
30–44	1,421 (87.4%)	205 (12.6%)	1,626	0.78	0.63–0.98	0.034
45–59	1,113 (89.1%)	136 (10.9%)	1,249	0.68	0.53–0.87	0.003
60+	379 (91.3%)	36 (8.7%)	415	0.55	0.38–0.82	0.003
Ethnicity						
Ethnic minority	460 (86.8%)	70 (13.27%)	530	1	-	-
White	3,520 (87.7%)	494 (12.3%)	4,014	1.00	0.76–1.33	0.991
Place of birth						
Other	875 (87.2%)	128 (12.8%)	1,003	1	-	-
UK	3,105 (87.7%)	436 (12.3%)	3,541	1.05	0.84–1.31	0.684
Country of residence during lookback period						
England	3,347 (87.4%)	482 (12.6%)	3,829	1	-	-
Other	633 (88.5%)	82 (11.5%)	715	0.93	0.72–1.19	0.553
Education level during lookback period						
Below degree	1,692 (87.7%)	238 (12.3%)	1,930	1	-	-
Degree & above	2,287 (87.5%)	326 (12.5%)	2,613	1.00	0.83–1.20	0.998
Employment status during lookback period						
Not employed	935 (88.2%)	125 (11.8%)	1,060	1	-	-
Employed	3,035 (87.4%)	438 (12.6%)	3,473	1.05	0.84–1.32	0.648
Living situation during lookback period						
Does not live with partner	2,762 (87.4%)	399 (12.6%)	3,161	1	-	-
Lives with partner	1,218 (88.1%)	165 (11.9%)	1,383	1.06	0.87–1.30	0.568
Number of new partners during lookback period						
0	2,079 (88.5%)	269 (11.5%)	2,348	1	-	-
1–2	962 (88.0%)	131 (12.0%)	1,093	1.04	0.83–1.31	0.714
3+	938 (85.1%)	164 (14.9%)	1,102	1.38	1.09–1.74	0.007
Chemsex in lookback period						
No chemsex	3,856 (87.6%)	544 (12.4%)	4,400	1	-	-
Used chemsex	124 (86.1%)	20 (13.9%)	144	0.95	0.58–1.57	0.844

1. Adjusted for sociodemographic and behavioural variables and including an interaction term to determine whether lookback period modified effect of HIV-PrEP use on respiratory symptoms

### The association between recent HIV-PrEP use and having a COVID-19 positive test

We then investigated the association between HIV-PrEP use and having a COVID-19 positive test at different periods of the pandemic. Of those 970 study participants in the three surveys reporting use of HIV-PrEP, 4.5% (n=44) reported a positive COVID-19 test and 95.5% (n=926) reported no positive COVID-19 test when asked about the most recent lookback period to the survey they were completing. Of the remaining 3,565 study participants who reported no use of HIV-PrEP, 3.3% (n=116) reported a positive COVID-19 test and 96.7% (n=3,449) reported no positive COVID-19 test when asked about the most recent lookback period to the survey they were completing.

After adjusting for sociodemographic and behavioural variables, we identified no significant association between HIV-PrEP use and testing positive for COVID-19 (aOR=1.41, CI=0.99–2.01.99.01, p=0.056) (S2 Table). However subsequent analysis revealed evidence of an interaction, such that the magnitude of the effect of HIV-PrEP use on having a positive COVID-19 test changed significantly across lookback periods (interaction p<0.0001). The results from the analysis including the interaction term are presented in Table 5. In P1 there was no association between HIV-PrEP use and a COVID-19 positive test (aOR=1.11, CI=0.51–3.98.51.98, p=0.868). However, as the pandemic progressed, this positive association increased substantially, such that participants who reported taking HIV-PrEP in P2 and P3 were significantly more likely to have had a positive COVID-19 test than those who did not report taking HIV-PrEP (P2: aOR=4.26, CI=1.21–14.97.21.97 p=0.023; P3: aOR-9.02, CI= 2.69–30.31.69.31, p<0.001).

**Table 5:** The association between COVID-19 confirmed by test (outcome) and HIV-PrEP use among men and gender diverse people who have sex with men during three study periods during the first year of the pandemic (main effects, including interaction term)

	No COVID-19 positive test	COVID-19 positive test	Total	adjusted Odds Ratio^1^	95% Confidence Interval	*P* value
HIV-PrEP use in lookback period (interaction term included between these two main effects)						
No HIV-PrEP in lookback period 1	1,485 (99.1%)	13 (0.9%)	1,498	1	-	-
Used HIV-PrEP in lookback period 1	306 (99.0%)	3 (1.0%)	309	1.11	0.31–3.98	0.868
No HIV-PrEP in lookback period 2	979 (96.5%)	35 (3.5%)	1,014	1	-	-
Used HIV-PrEP in lookback period 2	331 (95.7%)	15 (4.3%)	346	4.26	1.21–14.97	0.023
No HIV-PrEP in lookback period 3	985 (93.5%)	68 (6.5%)	1,053	1	-	-
Used HIV-PrEP in lookback period 3	289 (91.7%)	26 (8.3%)	315	9.02	2.69–30.31	< 0.001
Age at time of taking survey						
16–29	1,185 (94.6%)	68 (5.4%)	1,253	1	-	-
30–44	1,571 (96.6%)	55 (3.4%)	1,626	0.60	0.41-0.41.41.41 0.88	0.009
45–59	1,221 (97.8%)	28 (2.2%)	1,249	0.41	0.26–0.67	< 0.001
60+	406 (97.8%)	9 (2.2%)	415	0.37	0.18–0.78	0.009
Ethnicity						
Ethnic minority	500 (94.3%)	30 (5.7%)	530	1	-	-
White	3,884 (96.8%)	130 (3.2%)	4,014	0.59	0.38–0.92	0.020
Place of birth						
Other	966 (96.3%)	37 (3.7%)	1,003	1	-	-
UK	3,418 (96.5%)	123 (3.5%)	3,541	1.17	0.78–1.74	0.448
Country of residence during lookback period						
England	3,683 (96.2%)	146 (3.8%)	3,829	1	-	-
Other	701 (98.0%)	14 (2.0%)	715	0.49	0.28–0.86	0.013
Education level during lookback period						
Below degree	1,861 (96.4%)	69 (3.6%)	1,930	1	-	-
Degree & above	2,522 (96.5%)	91 (3.5%)	2,613	0.96	0.69–1.35	0.826
Employment status during lookback period						
Not employed	1,019 (96.1%)	41 (3.9%)	1,060	1	-	-
Employed	3,354 (96.6%)	119 (3.4%)	3,473	0.92	0.62–1.36	0.673
Living situation during lookback period						
Does not live with partner	3,042 (96.2%)	119 (3.8%)	3,161	1	-	-
Lives with partner	1,342 (97.0%)	41 (3.0%)	1,383	1.02	0.70–1.51	0.902
Number of new partners during lookback period						
0	2,282 (97.2%)	66 (2.8%)	2,348	1	-	-
1–2	1,054 (96.4%)	39 (3.6%)	1,093	1.02	0.67–1.55	0.937
3+	1,047 (95.0%)	55 (5.0%)	1,102	1.31	0.88–1.97	0.184
Chemsex in lookback period						
No chemsex	4,244 (96.5%)	156 (3.5%)	4,400	1	-	-
Used chemsex	140 (97.2%)	4 (2.8%)	144	0.64	0.23–1.80	0.396

1. Adjusted for sociodemographic and behavioural variables and including an interaction term to determine whether lookback period modified effect of HIV-PrEP use on COVID-19

## Discussion

### Principle findings

Using data from three large cross-sectional community based online surveys, we examined how reported COVID-19 varied according to use of HIV-PrEP among men and gender diverse people who have sex with men in the UK. We found no evidence that HIV-PrEP use protected against COVID-19 among participants. Indeed, we identified that participants who took HIV-PrEP at various stages of the COVID-19 pandemic were more likely to report a recent positive COVID-19 test result than participants who did not take HIV-PrEP. This positive association increased across the first year of the pandemic, with those participants taking HIV-PrEP in P2 and P3 having around four and nine times the odds of reporting COVID-19 than those who had not taken HIV-PrEP. However, whilst participants who reported using HIV-PrEP were increasingly more likely to receive a COVID-19 positive test result, the same group were less likely to report experiencing symptoms of a respiratory infection towards the end of the first year of the COVID-19 pandemic.

### Strengths and limitations

Our study takes place in the context of the first year of the COVID-19 pandemic and the early stages of the PrEP-era of HIV prevention in the UK. We used the same study protocol and similar recruitment methods for each survey resulting in broadly comparable population groups with similar sociodemographic profiles.

There are limitations to our study. As a cross-sectional survey, associations between variables can be bidirectional, and therefore it cannot be definitively concluded whether use of HIV-PrEP preceded a COVID-19 diagnosis. Participants were recruited through social media and dating applications thereby excluding people who do not use these platforms and those who are not seeking new sexual partners or have limited internet access. Online recruitment can be vulnerable to deliberate data manipulation by bots and spam responses, however there was little evidence to indicate that this significantly influenced our findings. This was prevented by complex survey routing and visual checking of data during our quality inspection. Furthermore, a comparison with four subsequent RiiSH survey waves shows consistency in reporting that would not indicate such manipulation of data. We used a convenience sample so the results may not be generalisable to all men and gender diverse people who have sex with men living in the UK. The RiiSH participants are more commonly White, UK-born, and may be a more health literate group [23]. This research was conducted as a sub-analysis of a larger study and thus sample sizes, particularly for our outcome variables, are relatively small and thus some confidence intervals are relatively wide However, the strength of the association is supportive of a true association.

The findings of this study are influenced by several challenges associated with conducting research in the rapidly changing and dynamic landscape of a global pandemic. For example, we identified that the proportion of participants reporting a positive COVID-19 test result increased significantly across surveys. This will have been directly affected by fluctuations in guidance on social mixing, number of infections and COVID-19 testing methods and availability throughout the pandemic in addition to individual testing frequency. Whilst we did not measure these factors in this study, the UK Health Security Agency have published data on case numbers (Epidemiology of COVID-19 in England: January 2020 to December 2024 - GOV.UK (https://www.gov.uk/government/publications/epidemiology-of-covid-19-in-england/epidemiologyof-covid-19-in-england-january-2020-to-december-2024www.gov.uk)) and testing behaviours (COVID-19: general public testing behaviours - GOV.UK (https://www.gov.uk/government/publications/lfd-tests-how-and-why-they-were-usedduring-the-pandemic/covid-19-general-public-testing-behaviourshttps://www.gov.ukhttp://www.gov.uk)). It is possible that participants who used HIV-PrEP were more likely to have visited sexual health services than participants who did not use HIV-PrEP and required a COVID-19 test prior to their visit. If so, a greater proportion of HIV-PrEP users than non-HIV-PrEP users might have tested positive for COVID-19 despite being asymptomatic. This would be dependent on the service model and period of the pandemic.

Susceptibility to COVID-19 infection was also likely influenced by changes in the infectivity and symptom burden of different variants as well as individual vaccination status. The first approved COVID-19 vaccination was not deployed until 8 December 2020 and so data on vaccination was unavailable in Survey 1 and 2. Whilst vaccination was available during the time that Survey 3 was in field, roll out was not complete for all age groups. Surveillance reports corresponding to this time period (Weekly Flu and COVID-19 https://assets.publishing.service.gov.uk/media/60814be7e90e076aaca7d631/Weekly_Flu_and_COVID-19_report_w16.pdfreport_w16) show that the overall uptake for one dose of the COVID-19 vaccination was 44.7%. Whilst higher in older age-groups (over 80% in 50+ year olds), uptake was much lower in younger age groups which make up the majority of our study population (55.5% in 45-under 50 year olds, 35.1% in 40–45 year olds and 12.5% in under 40 year olds).

Compliance with public health guidance on social distancing will vary with individual perceptions of risk, and potentially between those using and not using HIV-PrEP. Perception of risk may also have fluctuated at different points in the pandemic. However, gathering data on broader perceptions of risk was beyond the scope of this study.

Our analysis focused on any HIV-PrEP use within each lookback period compared with no HIV-PrEP use within each lookback period. It is possible that there is heterogeneity in the antiviral activity of HIV-PrEP depending on frequency of use, which may have masked the potential anti-viral protective effective against COVID-19. However, our findings are consistent with other studies showing no protective effect of HIV-PrEP against COVID-19.

Respiratory symptoms may be caused by a number of viruses, so it is not possible to deterministically confirm whether respiratory symptoms reported in the surveys were caused by COVID-19. Surveillance reports from the first year of the COVID-19 pandemic showed huge drops in reporting of non-COVID-19 respiratory illnesses (National flu and COVID-19 surveillance reports - https://www.gov.uk/government/statistics/national-flu-and-covid-19-surveillance-reportsGOV.UK), thus it is reasonable to assume that a large proportion of respiratory symptoms at this time were caused by COVID-19.

### Comparisons to other studies

Our finding that participants who took HIV-PrEP at various stages of the first year of the COVID-19 pandemic in the UK were more likely to report a recent positive COVID-19 test result aligns with previous findings from Spain [9]. The study analysed the seroprevalence of SARS-CoV-2 among people who used HIV-PrEP in Spain in May to June 2020 and showed that seroprevalence was higher in people who used HIV-PrEP compared with those who had not used HIV-PrEP (15% vs. 9.2% respectively). Other studies looking at the effect of HIV-PrEP on COVID-19 incidence have shown inconclusive results (a randomised control trial in Spain, Bolivia and Venezuela of healthcare workers taking HIV-PrEP for COVID-19 [12]) or no differences (a cohort study in France looking at COVID-19 incidence rates comparing PrEP users and the general population [13]).

It is possible that the higher SARS-CoV-2 prevalence observed in the HIV-PrEP group could be linked to sexual behaviour. It is likely that HIV-PrEP use is a marker of more social mixing and therefore, a higher risk of COVID-19. A study of men and gender diverse people who have sex with men in the UK found positive associations between reported HIV-PrEP use and continuing sexual behaviour with casual partners during lockdown restrictions [24]. A qualitative study exploring barriers to sexual distancing suggest that an active sex life before COVID-19 was associated with continuing sexual risk behaviour during COVID-19 related social restrictions [25]. Given COVID-19 is transmitted through close contact, continued, or increased sexual behaviour and partner numbers would increase the chance of exposure and infection of COVID-19. Our results suggest continued higher-risk sexual behaviour during COVID-19 related restrictions for people who used HIV-PrEP, which may help explain the association with increased likelihood to report a positive COVID-19 diagnosis.

Our findings could also reflect the development and increase in lockdown fatigue as the pandemic progressed, resulting in more socialisation and higher risk sexual behaviour for some men and gender diverse people who have sex with men, particularly those using HIV-PrEP [25]. For example, studies examining behaviour during the initial period of COVID-19-related social restrictions observed large decreases in reported HIV-PrEP use. One study found 47.0% of HIV-PrEP users reported stopping HIV-PrEP during Belgium’s first lockdown alongside a reduction in sex with non-steady partners (59.1% to 8.9% during the first weeks of lockdown) [26]. Thus people who use HIV-PrEP appropriately tailored their HIV-PrEP use in line with sexual risk activity [27, 28]. Sexual risk behaviours among men and gender diverse people who have sex with men in the UK then fluctuated as COVID-19-related social restrictions changed [22, 29]. In keeping with this, we found an increase in reported HIV-PrEP use as social restrictions eased, perhaps representative of the increased ‘need’ for HIV-PrEP as higher-risk sexual behaviour increased during the summer and autumn of 2020. We did not observe a large decrease in HIV-PrEP use during the UK’s third national lockdown. An Australian study reported similar findings; despite an initial decrease in reported HIV-PrEP use as COVID-19 restrictions were introduced, there was a general upwards trend in reported use between July 2020–June 2021 [28].

Whilst participants who reported using HIV-PrEP were increasingly more likely to receive a COVID-19 positive test, the same group were less likely to report experiencing symptoms of a respiratory infection towards the end of the first year of the COVID-19 pandemic. These findings are in line with a study in Brazil which showed that the regular use of HIV-PrEP was associated with lower self-reporting of COVID-19-related symptoms during the first month of the COVID-19 outbreak in São Paulo [14]. Of note, the earlier mentioned seroprevalence study in Spain, identified no statistically significant difference in symptoms of COVID-19 amongst people using HIV-PrEP [9]. However, whilst non-significant, their findings did appear to show some small differences in relation to clinical manifestations which align with our findings. In those who tested positive for SARS-CoV-2 and were receiving HIV-PrEP, 57.4% manifested symptoms, compared with 78.3% in the control group, and symptom duration was shorter (9 days vs. 11.5 days). Similar findings were also reported in a study in the USA which noted a potential protective association of TDF-based HIV-PrEP and severe COVID-19 outcomes [15]. The study reported that those with recent TDF-based HIV-PrEP use were 29% less likely to be hospitalized for any reason and 14% less likely to be hospitalized for COVID-19 compared to those who had not taken HIV-PrEP.

There are several possible explanations for our findings. If HIV-PrEP use is a marker for more social mixing then it is possible that compared with those not using HIV-PrEP, those using HIV-PrEP had greater exposure to all respiratory infections (COVID-19 or non-COVID-19). Given that symptoms of respiratory viruses tend to be similar, we are unable to determine whether reported respiratory symptoms are related to COVID-19 or to other respiratory viruses. However, greater exposure to any respiratory virus might offer some protection against subsequent exposure to closely related viruses, leading to fewer reported symptoms in this group over time.

Alternatively, it is possible that HIV-PrEP might in some way directly ameliorate the severity of respiratory symptoms. Whilst the specific association between HIV-PrEP use and COVID-19 symptom severity has only been reported in a few studies (highlighted above), there is some evidence in the literature for a potential protective effect of tenofovir-based therapy against adverse COVID-19 outcomes. This includes computer simulations and in vitro studies investigating the potential mechanisms of action [30–34], an in vivo study demonstrating a reduction in severity and duration of clinical symptoms in Ferrets [35], and a randomised control which found a significant reduction in COVID-19 viral burden in patients with non-severe COVID-19 treated with TDF in France [16]. Further research would be beneficial to better understand this association.

## Conclusions

This sub-analysis of data generated from three RiiSH-COVID surveys adds to the international evidence base on the relationship between HIV-PrEP use and COVID-19 among men and gender-diverse people who have sex with men. While the study is constrained by its modest sample size (being a sub-analysis of a larger study) and the rapidly changing context of the first year of the COVID-19 pandemic, the consistency of our findings with other research strengthens their relevance. Our findings indicate that people who use HIV-PrEP were more likely to acquire COVID-19, with this positive association increasing across the first year of the pandemic likely reflecting increased social mixing. Interestingly, the same group were less likely to report respiratory symptoms over the first year of the COVID-19 pandemic, possibly reflecting some immune protection following exposures to respiratory viruses through more social mixing or as a direct impact of HIV-PrEP on symptom severity. However, future studies with larger, more diverse cohorts and longitudinal designs are needed to confirm these associations and explore whether HIV-PrEP use confers any biological protection against severe respiratory illness.

## Supplementary Information

Supplementary Material 1.

## Data Availability

Data requests to support the findings of this study can be considered by University College London (UCL) on reasonable request and with permission from the UK Health Security Agency (UKHSA). Requests can be directed to Prof Cath Mercer ([c.mercer@ucl.ac.uk](mailto: c.mercer@ucl.ac.uk)).

## Abbreviations

aOR: Adjusted odds ratio
ARV: Antiretrovirals
CI: Confidence interval
FTC: Emtricitabine
HIV: Human Immunodeficiency Virus
HIV-PrEP: Human Immunodeficiency Virus - Pre-Exposure Prophylaxis
IQR: Interquartile range
MERS: Middle East Respiratory Syndrome
P1: Lookback Period 1
P2: Lookback Period 2
P3: Lookback Period 3
RiiSH: Reducing inequalities in Sexual Health
SARS: Severe Acute Respiratory Syndrome
SARS-CoV-2: Severe Acute Respiratory Syndrome Coronavirus 2
S1: Survey 1
S2: Survey 2
S3: Survey 3
STI: Sexually Transmitted Infections
TDF: Tenofovir
UCL: University College London
UK: United Kingdom
WHO: World Health Organisation

## References

[1] World Health Organisation. *WHO director-general’s opening remarks at the media briefing on COVID-19*. 2020.

[2] GOV.UK, *Prime minister’s statement on coronavirus (COVID-19).* 2020.

[3] Copertino DC Jr., et al. Antiretroviral drug activity and potential for pre-exposure prophylaxis against COVID-19 and HIV infection. J Biomol Struct Dyn. 2022;40(16):7367–80. 33734021 10.1080/07391102.2021.1901144 PMC8448789

[4] Ford N, et al. Systematic review of the efficacy and safety of antiretroviral drugs against SARS, MERS or COVID-19: initial assessment. J Int AIDS Soc. 2020;23(4):e25489. 32293807 10.1002/jia2.25489 PMC7158851

[5] Boban M. Novel coronavirus disease (COVID-19) update on epidemiology, pathogenicity, clinical course and treatments. Int J Clin Pract. 2021;75(4):e13868. 33244856 10.1111/ijcp.13868 PMC7744921

[6] Chien M, et al. Nucleotide analogues as inhibitors of SARS-CoV-2 polymerase, a key drug target for COVID-19. J Proteome Res. 2020;19(11):4690–7. 32692185 10.1021/acs.jproteome.0c00392 PMC7640960

[7] Del Amo J, et al. Incidence and severity of COVID-19 in HIV-positive persons receiving antiretroviral therapy. Ann Intern Med. 2021;174(4):581–2. 33872541 10.7326/L20-1399

[8] Boulle A, et al. Risk factors for coronavirus disease 2019 (COVID-19) death in a population cohort study from the Western cape Province, South Africa. Clin Infect Dis. 2021;73(7):e2005–15. 32860699 10.1093/cid/ciaa1198 PMC7499501

[9] Ayerdi O, et al. Preventive efficacy of Tenofovir/Emtricitabine against severe acute respiratory syndrome coronavirus 2 among pre-exposure prophylaxis users. Open Forum Infect Dis. 2020;7(11):ofaa455. 33200081 10.1093/ofid/ofaa455 PMC7543639

[10] Pebody R. *1600 people taking PrEP in Scotland and Wales, almost all are gay men.* aidsmap, 2018. available: https://www.aidsmap.com/news/apr-2018/1600-people-taking-prep-scotland-and-wales-almost-all-are-gay-men

[11] DHSC, HIV drug PrEP to be available across England. 2020. available: https://www.gov.uk/government/news/hiv-drug-prep-to-be-available-across-england

[12] Polo R, et al. Daily Tenofovir disoproxil fumarate/emtricitabine and hydroxychloroquine for pre-exposure prophylaxis of COVID-19: a double-blind placebo-controlled randomized trial in healthcare workers. Clin Microbiol Infect. 2023;29(1):85–93. 35940567 10.1016/j.cmi.2022.07.006 PMC9352647

[13] Delaugerre C, et al. Severe acute respiratory syndrome coronavirus 2 seroprevalence among HIV-negative participants using tenofovir/emtricitabine-based preexposure prophylaxis in 2020: a substudy of the French National Agency for Research on AIDS and Viral Hepatitis PREVENIR and Inserm SAPRIS-Sero Cohorts. Open Forum Infect Dis. 2022. 10.1093/ofid/ofac188. 35791355 10.1093/ofid/ofac188 PMC9047207

[14] Fernandes DE, Ferreira PRA, Mastroianni Kirsztajn G. Pre-exposure prophylaxis during the SARS-CoV-2 pandemic: can PrEP prevent COVID-19-related symptoms? Epidemiol Infect. 2020;148:e231. 32981567 10.1017/S0950268820002253 PMC7556904

[15] Lea AN, et al. Human immunodeficiency virus status, Tenofovir exposure, and the risk of poor coronavirus disease 19 outcomes: real-world analysis from 6 united States cohorts before vaccine rollout. Clin Infect Dis. 2023;76(10):1727–34. 36861341 10.1093/cid/ciad084 PMC10209434

[16] Parienti JJ, et al. Effect of tenofovir disoproxil fumarate and emtricitabine on nasopharyngeal SARS-CoV-2 viral load burden amongst outpatients with COVID-19: a pilot, randomized, open-label phase 2 trial. EClinicalMedicine. 2021;38:100993. 34222849 10.1016/j.eclinm.2021.100993 PMC8235994

[17] Wayal S, et al. The acceptability and feasibility of implementing a bio-behavioral enhanced surveillance tool for sexually transmitted infections in england: mixed-methods study. JMIR Public Health Surveill. 2018;4(2):e52. 29728348 10.2196/publichealth.9010 PMC5960042

[18] Wayal S, et al. Association between knowledge, risk behaviours, and testing for sexually transmitted infections among men who have sex with men: findings from a large online survey in the united Kingdom. HIV Med. 2019;20(8):523–33. 31124278 10.1111/hiv.12753 PMC6771985

[19] StataCorp. *Stata Statistical Software: Release 15.* College Station. TX: StataCorp LLC; 2017.

[20] Pearson K. On the criterion that a given system of deviations from the probable in the case of a correlated system of variables is such that it can be reasonably supposed to have arisen from random sampling. Philos Mag. 1900;1:157–75.

[21] Hyndman I, et al. COVID-19 restrictions and changing sexual behaviours in HIV-negative MSM at high risk of HIV infection in London, UK. Sex Transm Infect. 2021;97(7):521–4. 33462118 10.1136/sextrans-2020-054768

[22] Brown JR et al. Changes in STI and HIV testing and testing need among men who have sex with men during the uk’s COVID-19 pandemic response. Sex Transm Infect, 2022: p. sextrans–2022. 10.1136/sextrans-2022-055429 PMC10313956 35863887

[23] Ogaz D, et al. COVID-19 infection and vaccination uptake in men and gender-diverse people who have sex with men in the UK: analyses of a large, online community cross-sectional survey (RiiSH-COVID) undertaken November–December 2021. BMC Public Health. 2023;23(1):829. 37147609 10.1186/s12889-023-15779-5 PMC10161154

[24] Nadarzynski T, et al. The impact of first UK-wide lockdown (March-June 2020) on sexual behaviours in men and gender diverse people who have sex with men during the COVID-19 pandemic: a cross-sectional survey. Arch Sex Behav. 2023. 10.1007/s10508-022-02458-6. 36344786 10.1007/s10508-022-02458-6 PMC9640839

[25] de Vries DC, et al. Barriers and motives for complying with sexual distancing among men who have sex with men during the first COVID-19 pandemic lockdown in amsterdam: A qualitative study. Sex Transm Dis. 2022;49(7):497–503. 35404868 10.1097/OLQ.0000000000001636 PMC9196919

[26] Reyniers T, et al. Reduced sexual contacts with non-steady partners and less PrEP use among MSM in Belgium during the first weeks of the COVID-19 lockdown: results of an online survey. Sex Transm Infect. 2021;97(6):414–9. 33172917 10.1136/sextrans-2020-054756 PMC7656903

[27] Gillespie D, et al. PrEP use, sexual behaviour, and PrEP adherence among men who have sex with men living in Wales prior to and during the COVID-19 pandemic. AIDS Behav. 2022;26(8):2746–57. 35182283 10.1007/s10461-022-03618-4 PMC8857895

[28] Holt M, et al. Adjusting behavioural surveillance and assessing disparities in the impact of COVID-19 on gay and bisexual men’s HIV-related behaviour in Australia. AIDS Behav. 2022. 10.1007/s10461-022-03788-1. 35895148 10.1007/s10461-022-03788-1 PMC9326145

[29] Howarth AR, et al. ‘Stay at home …’: exploring the impact of the COVID-19 public health response on sexual behaviour and health service use among men who have sex with men: findings from a large online survey in the UK. Sex Transm Infect. 2022;98(5):346–52. 34544888 10.1136/sextrans-2021-055039 PMC8457994

[30] Yun Y, et al. Identification of therapeutic drugs against COVID-19 through computational investigation on drug repurposing and structural modification. J Biomed Res. 2020;34(6):458. 33122473 10.7555/JBR.34.20200044 PMC7718070

[31] Toor HG, et al. Computational drug re-purposing targeting the Spike glycoprotein of SARS-CoV-2 as an effective strategy to neutralize COVID-19. Eur J Pharmacol. 2021;890:173720. 33160938 10.1016/j.ejphar.2020.173720 PMC7644434

[32] Rahman MR, et al. Identification of potential antivirals against SARS-CoV-2 using virtual screening method. Inform Med Unlocked. 2021;23:100531. 33594342 10.1016/j.imu.2021.100531 PMC7874919

[33] Shah B, Modi P, Sagar SR. In silico studies on therapeutic agents for COVID-19: drug repurposing approach. Life Sci. 2020;252:117652. 32278693 10.1016/j.lfs.2020.117652 PMC7194845

[34] Clososki GC, et al. Tenofovir disoproxil fumarate: new chemical developments and encouraging in vitro biological results for SARS-CoV-2. J Braz Chem Soc. 2020;31:1552–6.

[35] Park S-J, et al. Antiviral efficacies of FDA-approved drugs against SARS-CoV-2 infection in ferrets. mBio. 2020. 10.1128/mbio.01114-20. 32444382 10.1128/mBio.01114-20 PMC7244896

