# Supplementary material for: PrEPped for COVID? The association between HIV-PrEP use and COVID-19 among men and gender diverse people who have sex with men: findings from three large online surveys in the United Kingdom
Source: BMC Public Health. 2026 Jan 22;26:624. doi: 10.1186/s12889-025-26127-0 (PMC12911014; doi:10.1186/s12889-025-26127-0)
Supplement: Supplementary file 1 — Supplementary Material 1. [file 12889_2025_26127_MOESM1_ESM.docx]

**S1 Table: The association between respiratory symptoms and HIV-PrEP use among men and gender diverse people who have sex with men (excluding interaction term)**

|  | **No respiratory symptoms** | **Respiratory symptoms** | **Total** | **unadjusted Odds Ratio** | **95% Confidence Interval** | **P value** | **adjusted Odds Ratio** | **95% Confidence Interval** | **P value** |
| --- | --- | --- | --- | --- | --- | --- | --- | --- | --- |
| **HIV-PrEP use in lookback period** | | | | | | | | | |
| **No HIV-PrEP in lookback period** | **3,134 (87.9%)** | **431 (12.1%)** | **3,565** | **1** | **-** | **-** | **1** | **-** | **-** |
| **Used HIV-PrEP in lookback period** | **839 (86.5%)** | **131 (13.5%)** | **970** | **1.14** | **0.92-1.40** | **0.236** | **1.08** | **0.86-1.35** | **0.509** |
| **Lookback period** | | | | | | | | | |
| **Lookback period 1** | **1,567 (86.3%)** | **248 (13.7%)** | **1,815** | **1** | **-** | **-** | **1** | **-** | **-** |
| **Lookback period 2** | **1,201 (88.2%)** | **160 (11.8%)** | **1,361** | **0.84** | **0.68-1.04** | **0.112** | **0.77** | **0.62-0.97** | **0.023** |
| **Lookback period 3** | **1,212 (88.6%)** | **156 (11.4%)** | **1,368** | **0.81** | **0.28-0.72** | **0.058** | **0.79** | **0.63-0.98** | **0.030** |
| **Age at time of taking survey** | | | | | | | | | |
| **16-29** | **1,066 (85.1%)** | **187 (14.9%)** | **1,253** | **1** | **-** | **-** | **1** | **-** | **-** |
| **30-44** | **1,421 (87.4%)** | **205 (12.6%)** | **1,626** | **0.82** | **0.66-1.02** | **0.073** | **0.79** | **0.63-0.99** | **0.039** |
| **45-59** | **1,113 (89.1%)** | **136 (10.9%)** | **1,249** | **0.70** | **0.54-0.88** | **0.003** | **0.69** | **0.53-0.88** | **0.003** |
| **60+** | **379 (91.3%)** | **36 (8.7%)** | **415** | **0.54** | **0.37-0.79** | **0.001** | **0.56** | **0.38-0.83** | **0.004** |
| **Ethnicity** | | | | | | | | | |
| **Ethnic minority** | **460 (86.8%)** | **70 (13.27%)** | **530** | **1** | **-** | **-** | **1** | **-** | **-** |
| **White** | **3,520 (87.7%)** | **494 (12.3%)** | **4,014** | **0.92** | **0.71-1.21** | **0.555** | **0.99** | **0.75-1.31** | **0.953** |
| **Place of birth** | | | | | | | | | |
| **Other** | **875 (87.2%)** | **128 (12.8%)** | **1,003** | **1** | **-** | **-** | **1** | **-** | **-** |
| **UK** | **3,105 (87.7%)** | **436 (12.3%)** | **3,541** | **0.96** | **0.78-1.19** | **1.704** | **1.05** | **0.84-1.31** | **0.699** |
| **Country of residence during lookback period** | | | | | | | | | |
| **England** | **3,347 (87.4%)** | **482 (12.6%)** | **3,829** | **1** | **-** | **-** | **1** | **-** | **-** |
| **Other** | **633 (88.5%)** | **82 (11.5%)** | **715** | **0.90** | **0.70-1.15** | **0.405** | **0.93** | **0.72-1.19** | **0.552** |
| **Education level during lookback period** | | | | | | | | | |
| **Below degree** | **1,692 (87.7%)** | **238 (12.3%)** | **1,930** | **1** | **-** | **-** | **1** | **-** | **-** |
| **Degree & above** | **2,287 (87.5%)** | **326 (12.5%)** | **2,613** | **1.01** | **0.85-1.21** | **0.884** | **0.99** | **0.83-1.20** | **0.948** |
| **Employment status during lookback period** | | | | | | | | | |
| **Not employed** | **935 (88.2%)** | **125 (11.8%)** | **1,060** | **1** | **-** | **-** | **1** | **-** | **-** |
| **Employed** | **3,035 (87.4%)** | **438 (12.6%)** | **3,473** | **1.08** | **0.87-1.33** | **0.479** | **1.06** | **0.85-1.33** | **0.605** |
| **Living situation during lookback period** | | | | | | | | | |
| **Does not live with partner** | **2,762 (87.4%)** | **399 (12.6%)** | **3,161** | **1** | **-** | **-** | **1** | **-** | **-** |
| **Lives with partner** | **1,218 (88.1%)** | **165 (11.9%)** | **1,383** | **0.94** | **0.77-1.14** | **0.515** | **1.05** | **0.86-1.30** | **0.577** |
| **Number of new partners during lookback period** | | | | | | | | | |
| **0** | **2,079 (88.5%)** | **269 (11.5%)** | **2,348** | **1** | **-** | **-** | **1** | **-** | **-** |
| **1-2** | **962 (88.0%)** | **131 (12.0%)** | **1,093** | **1.05** | **0.84-1.31** | **0.652** | **1.05** | **0.84-1.32** | **0.662** |
| **3+** | **938 (85.1%)** | **164 (14.9%)** | **1,102** | **1.35** | **1.10-1.67** | **0.005** | **1.38** | **1.09-1.73** | **0.007** |
| **Chemsex in lookback period** | | | | | | | | | |
| **No chemsex** | **3,856 (87.6%)** | **544 (12.4%)** | **4,400** | **1** | **-** | **-** | **1** | **-** | **-** |
| **Chemsex** | **124 (86.1%)** | **20 (13.9%)** | **144** | **1.14** | **0.71-1.85** | **0.585** | **0.97** | **0.59-1.59** | **0.893** |

**S2 Table: The association between COVID-19 confirmed by test and HIV-PrEP use among men and gender diverse people who have sex with men (excluding interaction term)**

|  | **No COVID-19 positive test** | **COVID-19 positive test** | **Total** | **unadjusted Odds Ratio** | **95% Confidence Interval** | **P value** | **adjusted Odds Ratio** | **95% Confidence Interval** | **P value** |
| --- | --- | --- | --- | --- | --- | --- | --- | --- | --- |
| **HIV-PrEP use in lookback period** | | | | | | | | | |
| **No HIV-PrEP in lookback period** | **3,449 (96.7%)** | **116 (3.3%)** | **3,565** | **1** | **-** | **-** | **1** | **-** | **-** |
| **Used HIV-PrEP in lookback period** | **926 (95.5%)** | **44 (4.5%)** | **970** | **1.41** | **0.99-2.01** | **0.056** | **1.23** | **0.83-1.81** | **0.298** |
| **Lookback period** | | | | | | | | | |
| **Lookback period 1** | **1,799 (99.1%)** | **16 (0.9%)** | **1,815** | **1** | **-** | **-** | **1** | **-** | **-** |
| **Lookback period 2** | **1,311 (96.3%)** | **50 (3.7%)** | **1,361** | **4.29** | **2.43-7.56** | **<0.0001** | **3.91** | **2.19-6.95** | **<0.0001** |
| **Lookback period 3** | **1,274 (93.3%)** | **94 (6.9%)** | **1,368** | **8.30** | **4.86-14.16** | **<0.0001** | **8.30** | **4.83-14.24** | **<0.0001** |
| **Age at time of taking survey** | | | | | | | | | |
| **16-29** | **1,066 (85.1%)** | **187 (14.9%)** | **1,253** | **1** | **-** | **-** | **1** | **-** | **-** |
| **30-44** | **1,421 (87.4%)** | **205 (12.6%)** | **1,626** | **0.61** | **0.42-0.88** | **0.008** | **0.60** | **0.41-0.88** | **0.009** |
| **45-59** | **1,113 (89.1%)** | **136 (10.9%)** | **1,249** | **0.40** | **0.26-0.63** | **<0.0001** | **0.41** | **0.26-0.67** | **<0.0001** |
| **60+** | **379 (91.3%)** | **36 (8.7%)** | **415** | **0.39** | **0.19-0.78** | **0.008** | **0.37** | **0.18-0.78** | **0.009** |
| **Ethnicity** | | | | | | | | | |
| **Ethnic minority** | **460 (86.8%)** | **70 (13.27%)** | **530** | **1** | **-** | **-** | **1** | **-** | **-** |
| **White** | **3,520 (87.7%)** | **494 (12.3%)** | **4,014** | **0.56** | **0.37-0.84** | **0.005** | **0.60** | **0.38-0.92** | **0.020** |
| **Place of birth** | | | | | | | | | |
| **Other** | **875 (87.2%)** | **128 (12.8%)** | **1,003** | **1** | **-** | **-** | **1** | **-** | **-** |
| **UK** | **3,105 (87.7%)** | **436 (12.3%)** | **3,541** | **0.94** | **0.65-1.37** | **0.744** | **1.17** | **0.78-1.74** | **0.448** |
| **Country of residence during lookback period** | | | | | | | | | |
| **England** | **3,347 (87.4%)** | **482 (12.6%)** | **3,829** | **1** | **-** | **-** | **1** | **-** | **-** |
| **Other** | **633 (88.5%)** | **82 (11.5%)** | **715** | **0.50** | **0.29-0.88** | **0.015** | **0.49** | **0.28-0.86** | **0.013** |
| **Education level during lookback period** | | | | | | | | | |
| **Below degree** | **1,692 (87.7%)** | **238 (12.3%)** | **1,930** | **1** | **-** | **-** | **1** | **-** | **-** |
| **Degree & above** | **2,287 (87.5%)** | **326 (12.5%)** | **2,613** | **0.97** | **0.71-1.34** | **0.867** | **0.96** | **0.69-1.35** | **0.829** |
| **Employment status during lookback period** | | | | | | | | | |
| **Not employed** | **935 (88.2%)** | **125 (11.8%)** | **1,060** | **1** | **-** | **-** | **1** | **-** | **-** |
| **Employed** | **3,035 (87.4%)** | **438 (12.6%)** | **3,473** | **0.88** | **0.61-1.27** | **0.496** | **0.92** | **0.62-1.36** | **0.672** |
| **Living situation during lookback period** | | | | | | | | | |
| **Does not live with partner** | **2,762 (87.4%)** | **399 (12.6%)** | **3,161** | **1** | **-** | **-** | **1** | **-** | **-** |
| **Lives with partner** | **1,218 (88.1%)** | **165 (11.9%)** | **1,383** | **0.78** | **0.54-1.12** | **0.179** | **1.02** | **0.70-1.51** | **0.902** |
| **Number of new partners during lookback period** | | | | | | | | | |
| **0** | **2,079 (88.5%)** | **269 (11.5%)** | **2,348** | **1** | **-** | **-** | **1** | **-** | **-** |
| **1-2** | **962 (88.0%)** | **131 (12.0%)** | **1,093** | **1.28** | **0.86-1.91** | **0.230** | **1.02** | **0.67-1.54** | **0.941** |
| **3+** | **938 (85.1%)** | **164 (14.9%)** | **1,102** | **1.82** | **1.26-2.62** | **0.001** | **1.31** | **0.88-1.97** | **0.184** |
| **Chemsex in lookback period** | | | | | | | | | |
| **No chemsex** | **3,856 (87.6%)** | **544 (12.4%)** | **4,400** | **1** | **-** | **-** | **1** | **-** | **-** |
| **Chemsex** | **124 (86.1%)** | **20 (13.9%)** | **144** | **0.78** | **0.28-2.13** | **0.624** | **0.64** | **0.23-1.80** | **0.395** |
